# Antibiotic prescribing in inpatient and outpatient settings in Iran: a systematic review and meta-analysis study

**DOI:** 10.1186/s13756-021-00887-x

**Published:** 2021-01-14

**Authors:** Ehsan Nabovati, Zhila TaherZadeh, Saeid Eslami, Ameen Abu-Hanna, Reza Abbasi

**Affiliations:** 1 Health Information Management Research Center, Kashan University of Medical Sciences, Kashan, Iran; 2 Department of Health Information Management and Technology, School of Allied Health Professions, Kashan University of Medical Sciences, Kashan, Iran; 3 Targeted Drug Delivery Research Center, School of Pharmacy, Mashhad University of Medical Sciences, Mashhad, Iran; 4 Department of Medical Informatics, Faculty of Medicine, Mashhad University of Medical Sciences, Mashhad, Iran; 5 Pharmaceutical Research Center, Pharmaceutical Research Institute, Mashhad University of Medical Sciences, Mashhad, Iran; 6 Department of Medical Informatics, Amsterdam UMC - Location AMC, University of Amsterdam, Amsterdam, The Netherlands

**Keywords:** Antibiotic prescribing, Antibiotic utilization, Developing countries, Middle income countries, Iran

## Abstract

**Background:**

Antibiotic prescribing is common worldwide. There are several original studies about antibiotic prescribing in the healthcare setting of Iran reporting different levels of prescribing. The aim of this systematic review and meta-analysis was to determine the prevalence of antibiotic prescribing in both inpatient and outpatient settings in Iran, an example of a developing country.

**Methods:**

To identify published studies on antibiotic prescribing, databases such as ISI, Scopus, PubMed, Google Scholar, and Electronic Persian were searched in Iran till January 2020. Eligible studies were those analyzing original data on the prescription and use of antibiotics in outpatient or inpatient settings in Iran. Moreover, all studies that used an intervention to improve antibiotic prescribing were included. The quality of the included studies was assessed using self-administered quality assessment criteria. The meta-analysis of prevalence of antibiotic prescribing was conducted based on the meta-analysis of observational studies in epidemiology guidelines. To calculate pooled rates, the random-effects model was used.

**Results:**

A total of 54 studies (39 outpatients and 15 inpatients) were included in this study. The median of antibiotic prescribing in the outpatient and inpatient settings accounted for 45.25% and 68.2% of patients, respectively. The results of meta-analysis also showed that the antibiotic prescribing accounted for 45% of prescriptions in outpatient settings and 39.5%, 66%, and 75.3% of patients in all wards, pediatrics wards, and ICU wards of inpatient settings, respectively. The most commonly prescribed antibiotic classes in outpatient settings were penicillins, cephalosporins, and macrolides, while in inpatient settings, these were cephalosporins, penicillins, and carbapenems. There were seven studies using interventions to improve antibiotic prescribing pattern. It should be mentioned that intervention in a study had a statistically significant effect on improving antibiotic prescribing (*p* < .05).

**Conclusion:**

Prevalence of antibiotic prescribing in Iran is high. Our findings highlight the need for urgent action to improve prescription practices. It seems that developing a national plan to improve antibiotic prescribing is necessary.

## Background

Prescribing and use of antibiotics have spread worldwide. These medications are among the most frequently used and expensive drugs for patients as well as health care organizations [1]. Although the use of antibiotics is helpful in the treatment of patients, their irrational, excessive use has become a major concern; therefore, it has led to the spread of antibiotic resistance, one of the greatest threats to human health. Antibiotic resistance may lead to delay in providing effective care, increased costs, and even death [2–5]. One of the most important reasons for antibiotic resistance is inappropriate, excessive use of antibiotics [6, 7].

Monitoring the patterns and rates of antibiotic prescribing are among the recommended strategies to prevent their overuse [8]. In addition, determining the prevalence of antibiotic prescribing is one of the main criteria in evaluating the antibiotics status [9]. According to the World Health Organization (WHO), the ideal prevalence for antibiotic prescribing is 20–26.8% of prescriptions [1, 11]. The antibiotic prescribing rate is increasing and often exceeds the WHO recommendation threshold in various developed and developing countries such as USA, many European countries (such as France, Spain, Portugal, Cyprus, Iceland, Greece, and Czech Republic), Asian, and African countries (such as China, Thailand, Saudi Arabia, Jordan, and Egypt) [11–18].

Many studies estimated the rate of antibiotic prescribing in inpatient and outpatient settings in different countries and reported different estimates [19–25]. However, there are few studies that have systematically reviewed the rate of antibiotic prescribing worldwide [18, 26, 27]. The results of two systematic review and meta-analysis studies in China showed that the overall rate of antibiotic prescribing in healthcare settings and in patients with upper respiratory tract infection (URTI) was high [18, 26]. The results of another one revealed that the rate of antibiotic prescribing in pediatrics hospitals in countries with poor resources was high [27]. Antibiotic usage is high in Iran which is a developing country. Some studies reported antibiotic prescribing prevalence of 45–72% in inpatients and outpatients in this country [28–30]. Several studies have been conducted to evaluate the rate of antibiotic prescribing in different geographical areas, healthcare settings, and even in a number of patients [30, 33–36] and each of which reported different rates. Therefore, it is important to perform a systematic review studies appear to be helpful in planning and controlling the use and prescription of these medications by providing a summary of the evidence and an overview of antibiotic prescribing in healthcare settings. This supports decision making pertaining to health about antibiotic prescribing. Thus, this systematic review and meta-analysis study aimed at determining the prevalence of antibiotic prescribing in different settings in Iran.

## Methods

### Search strategy

A comprehensive search strategy was developed using terms and MeSH terms related to antibiotic (e.g. antibiotic, anti-infective agents, antimicrobial, and antibacterial), prescribing (e.g. prescription, prescribe, administer, dispense, consumption, therapy, and Treat), and Iran (e.g. Iran, Iranian, Farsi, Persian).

Electronic databases (i.e. ISI, Scopus, and MEDLINE/PubMed) were searched using customized search strategies on Jan 2020. Persian electronic databases, including MagIran and SID (Scientific Information Database), were searched using Persian terms which are similar to the above-mentioned ones. Google Scholar was also searched using Persian search terms, to avoid missing relevant papers. Finally, the list of references in all retrieved papers was reviewed to identify extra relevant studies.

### Inclusion and exclusion criteria

The original studies included were those investigating antibiotic prescribing rate in patients’ prescriptions or hospital patients’ records, those using an intervention to improve antibiotic prescribing pattern and the ones conducted in either inpatient or outpatient settings in Iran and published in Persian or English. Due to the necessity of prescribing antibiotics in surgery, dentistry, and burn patients, the studies on these populations were not included. In addition, conference papers, letters, opinions, and dissertations were also excluded.

### Review procedure and data extraction

One of the reviewers searched the databases (R.A). Screening the title and abstract of potentially relevant papers were carried out by two independent reviewers (E.N, R.A). Any potential conflict about the inclusion of papers was discussed by them and after reaching a consensus, they made an appropriate decision. Subsequently, the full text of the included papers was retrieved to fulfil the aims of this review, and if not available, the full-texts were requested from the authors via email.

After reviewing the full-text for each included paper, the following information was extracted: authors, year of study, region, setting, sample size, unit of analysis, percentage of antibiotic prescribing (per prescription or medical record), the number of antibiotics in each prescription (one, two, and more than two antibiotics), and antibiotic classes and names. In the case of interventional studies, in addition to the above mentioned information, the type of study, the type of intervention used, and its effects were also extracted. Since patients’ age and gender, type of disease, type of insurance, and type of cost payment have not been investigated in most of the included studies, we could not consider this type of data in our analysis.

### Quality assessment of the included studies

The methodological quality of the included studies was evaluated by a self-administered checklist based on related studies [18, 37] and approved by three specialists (Pharmacology, medical informatics, and health information management) (Table 1).

**Table 1:** Quality assessment criteria for the included studies

Quality assessment criteria	Score
Study subjects have been described	1
Aims/objectives of study have been clearly stated	1
Data collection method has been clearly described	1
Type of healthcare setting has been mentioned	1
The methods of sampling and calculation of sample size have been explained	1
Percentage of antibiotic prescribing has been specified	1
Groups receiving prescribed antibiotics have been specified	1
Names of prescribed antibiotics have been specified	1
Number of prescribed antibiotics in each prescription has been specified (one antibiotic, 2 antibiotics, and more than 2 antibiotics)	1
Limitations of study have been stated	1
Maximum score	10

Total quality scores ranged from 0 to 10 (0–4 points = poor, 5–7 points = moderate, 8–10 points = high). Two independent reviewers scored the quality of each study according to the mentioned criteria and the third reviewer resolved potential discrepancies.

### Statistical analysis

The median and interquartile range (IQR) of antibiotic prescribing rates were calculated. Subgroup analyses were conducted based on the healthcare setting type (inpatient and outpatient). To standardize the meta-analysis methodology, the rates of antibiotic prescribing were obtained. Data were analyzed using Comprehensive Meta-Analysis (CMA) software (Version 2.0). The meta-analysis was conducted based on the meta-analysis of observational studies in epidemiology guidelines [38]. Interventional studies were excluded for meta-analysis. Pooled rates were calculated with a 95% confidence interval (CI) using a random-effects model. For publication bias, Egger’s weighted regression method was used.

## Results

### Literature search results

**Fig. 1:**
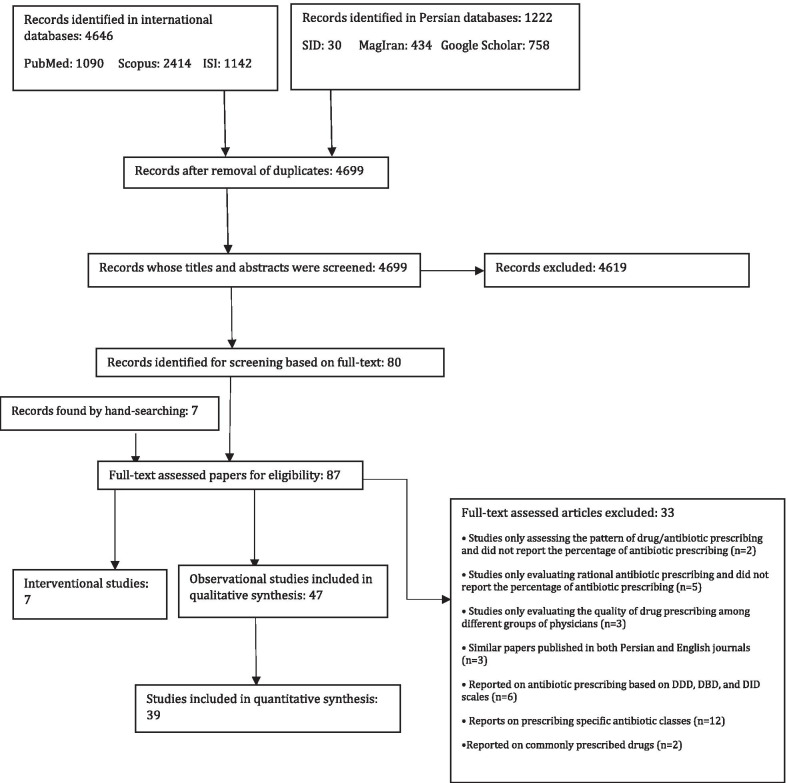
Flow diagram of the literature search and study selection

Figure 1 shows the flow diagram of the literature search. The electronic literature search led to the identification of 5868 published papers. After excluding duplicates, 4699 unique papers remained. After reviewing the titles of the papers as well as their abstracts and also considering the inclusion and exclusion criteria, 80 papers were selected for full-text review. Furthermore, by hand-searching in Google Scholar and the reference lists of the included papers, 7 additional related papers were identified. After a detailed full-text review of 87 papers, 33 papers were excluded because they reported antibiotic prescribing based on the defined daily dose (DDD), defined daily dose per 100 Inhabitants per day (DID), or defined daily dose per bed day (DBD) scales [33, 35, 39–42], or only assessed prescriptions containing antibiotics and did not report the ratio of antibiotic-containing prescriptions to all prescriptions [43, 44]. Finally, a total of 54 papers were selected to be included in this study.

### General characteristics of the included studies

The included studies were conducted from 1995 to 2016. In total, 39 (72%) studies were conducted in outpatient settings and 15 (28%) in inpatient settings. Out of 39 studies in outpatient settings, 7 (18%) studies evaluated the effects of interventions on antibiotic prescription [45–51].

### Quality of the included studies

The quality assessment of the included studies showed that none of them fulfilled all the quality criteria. Twelve (22%) studies were of high quality, 35 (65%) were of moderate quality, and 7 (13%) were of poor quality. Only 18 studies (33%) listing their limitations.

### Findings obtained from the interventional studies

All of the interventional studies were conducted in outpatient settings during 1995–2012. Since there was difference in the percentage of antibiotic prescribing in before and after of interventions, the results of interventional studies were reported separately from other outpatient studies (Table 2). All the interventions were educational [45–49], and in two studies [50, 51], both feedback and educational materials were used. The interventions used in these studies resulted in a relative improvement in antibiotic prescribing pattern; however, in one study, the effect of intervention was statistically significant (*p* < 0.05) [45].

**Table 2:** General characteristics of the interventional studies on antibiotic prescription

References	Region	Setting	Sample size/unit of analysis	Study type/duration	Intervention type	Percentage of the prescribed antibiotics	Effects of the intervention	The most commonly prescribed antibiotic classes (from most to least)	The most commonly prescribed antibiotics (from most to least)
Mohagheghi, et al. (1995–2001) [45]	Tehran	Outpatient	1,096,861 prescriptions	RCT/6 months	Short educational course for GPs	Before: in IG, 66.8% and in CG, 71.4% After: in IG, 66.1% and in CG, 74.8%	Antibiotic prescribing was less in IG than in CG (*p* < 0.05)	Penicillin, Sulfonamides, Cephalosporin, Macrolides, Metronidazole, Aminoglycosides	NM
Najaf Zare et al. [46]	Shiraz	Outpatient	119 GPs	Quasi experimental (before and after)/one year	Rational prescribing workshop (one day)	Before: 47.3% After: 46.4%	Relative improvement (*p* > 0.05)	NM	NM
Garjani et al. [47]	Tabriz	Outpatient	1135 prescriptions	RCT/One month	Educational intervention reviewing examples of prescriptions, principles of prescription writing, necessity of rational prescribing and use of drugs, impact of irrational use of drugs, common errors in prescribing, and rational use of injections, antibiotics, and glucocorticoids (why, where, how, and how long)	Before in both groups: 40.8% After: in IG, 38.9% and in CG, 37.2%	Relative improvement in prescribing (*p* > 0.05)	Penicillin, Cephalosporin, Aminoglycosides	NM
Ataei et al. [48]	Kermanshah	Outpatient	2040 prescriptions	Quasi experimental (Before and After)/6 months	Rational prescribing workshop	Before: 52.2% After: 47.6%	Less prescribing antibiotics (*p* > 0.05)	NM	NM
Esmaily [49]	East Azarbayjan	Outpatient	159 GPs	CRCT/3 day	Educational programs: (1) principles of prescription writing, (2) adverse reactions to drugs, (3) drug interactions, (4) injections, (5) antibiotic therapy, and (6) therapy with anti-inflammatory agents	Pretest (IG: 61%, CG: 59%)	No significant improvement (*p*-value: IG: .41 and CG: .39)	NM	NM
						Posttest (IG: 63%, CG: 60%)			
Sadeghi et al. [50]	Cheharmahal Bakhtiyari	Outpatient	50 physicians	Quasi experimental with an external control group	Feedback for patients with mean of the country, a book about rational prescribing medications, content of Iran’s drugs	Before: in IG1, 59.4% and in IG2, 58.5%	Relative improvement (*p* > 0.05)	NM	NM
						After: in IG1, 50.3% and in IG2, 59.38%			
Soleymani et al. [51]	Tehran	Outpatient	800 physicians	RCT/3 months	Four-armed randomized controlled trial: routinely conducting audit and feedback (RA&F), newly-designed audit and feedback (NA&F), printed educational materials (PEM) as well as a control arm	RA&F: 50.14%	None of the interventions were effective in reducing overall antibiotic use	NM	NM
						NA&F: 47.79%			
						PEM: 48.19%			
						Control: 47.05%			

*CG* control group, *CRCT* cluster randomized control trial, *GP* general practitioner, *IG* intervention group, *NM* not mentioned, *RCT* randomized control trial

### The prevalence of antibiotic prescription in inpatient healthcare settings

Among the reviewed studies, 15 (28%) ones were done in inpatient settings during 1997–2014. In two studies (13%) [52, 53], the unit of analysis was the prescriptions. In the other 13 studies (87%), the unit of analysis was patients or hospital patients' records, which were considered to be equal. Also, 3, 4, and 4 studies were done in all wards, pediatrics wards, and ICU wards, respectively. The minimum and maximum sample sizes were 104 and 17,668 patients, respectively. Most of the studies (80%) did not report the most commonly prescribed antibiotic classes. The median of antibiotic prescribing in inpatient setting accounted for 68.2% of patients. Cephalosporins, carbapenems, and penicillins were the most commonly prescribed antibiotic classes. Ceftriaxone, cefazolin, vancomycin, and clindamycin were the most commonly prescribed antibiotics (Table 3). Due to difference in the various inpatient settings (i.e. all wards, pediatrics, ICU, and emergency), the total meta-analysis was not performed but just conducted separately on similar settings. The meta-analysis results showed that in the studies pertaining to all wards, pediatrics wards, and ICU wards, antibiotics were prescribed for 39.5, 66, and 75.3% of patients, respectively (Additional file 1: Attachments).

**Table 3:** General characteristics of the studies conducted in inpatient settings

References	Region	Setting	Sample size	Percentage of antibiotics prescribed	The most commonly prescribed antibiotic classes (from most to least)	The most commonly prescribed antibiotics (from most to least)	Unit of analysis
Cheragh Ali et al. [52]	Tehran	Inpatient (all wards)	3117 prescriptions	39	NM	NM	Prescriptions
Shayan et al. [53]	Jahrom	Inpatient (all wards)	4969 prescriptions	56.63	NM	NM	
Hajebi et al. [54]	Tehran	Inpatient (all wards)	2137 hospital records	57	Cephalosporin, Penicillin, Carbapenems, Aminoglycosides	NM	Patients or hospital records
Tavallaee et al. [55]	Tehran	Inpatient (ICU)	119 patients	95.5	NM	Cefuroxime, Ceftriaxone, clindamycin, Cefazolin, Vancomycin	
Khodabakhshi et al. [56]	Golestan	Inpatient (all wards)	318 hospital records	69	NM	Ceftriaxone, Clindamycin, Cefazolin, Metronidazole, Gentamycin	
Khakshour et al. [57]	Bojnurd	Inpatient (pediatrics ward)	292 patients	78	NM	NM	
Mostafavi et al. [32]	Iran (All provinces)	Inpatient (pediatrics ward)	1506 patients	62.6	NM	NM	
Taghi Zadeh et al. [36]	Tabriz	Inpatient (ICU ward)	234 hospital records	82	Cephalosporin, Carbapenems, Penicillin, β-lactam, Aminoglycosides	Cefazolin, Cefepime, Ceftriaxone, Ceftazidime, Meropenem	
Alavi et al. [58]	Ahvaz	Inpatient (all wards)	9082 patients	34.34	NM	Ceftriaxone, Cloxacillin, Cefazolin, Gentamycin, Amikacin	
Rafati et al. [59]	Sari	Inpatient (ICU ward)	148 patients	68.2	NM	NM	
Abbasi et al. [60]	Urmia	Inpatient (pediatrics ward)	104 patients	85.6	NM	Ceftriaxone, Ceftizoxime	
Reihani et al. [61]	Mashhad	Inpatient (emergency ward)	540 patients	70.2	Cephalosporin, Macrolides, Carbapenems, Penicillin, Aminoglycosides	NM	
Fahimzad et al. [30]	Iran (17 hospitals from 15 cities)	Inpatient (neonatal and NICU wards)	366 patients	72	NM	Ampicillin, Vancomycin, Amikacin, Cefotaxime, Gentamycin,	
Fahimzad et al. [31]	Iran (16 Iranian pediatric hospitals)	Inpatient (pediatrics ward)	858 patients	67	NM	Ceftriaxone, Vancomycin, Cefotaxime, Ceftazidime, Metronidazole	
Sabour et al. [62]	Tehran	Nursing homes	170 hospital records	13.5	NM	NM	
			Median: 366	Median: 68.2	Most prescribed: Cephalosporin, Carbapenems, and Penicillins	Most prescribed: Ceftriaxone, Cefazolin, Vancomycin, and Clindamycin	
			IQR1: 170	IQR1: 56.63			
			IQR3: 2137	IQR3: 78			

*NM* not mentioned

### The prevalence of antibiotic prescription in outpatient healthcare settings

**Fig. 2:**
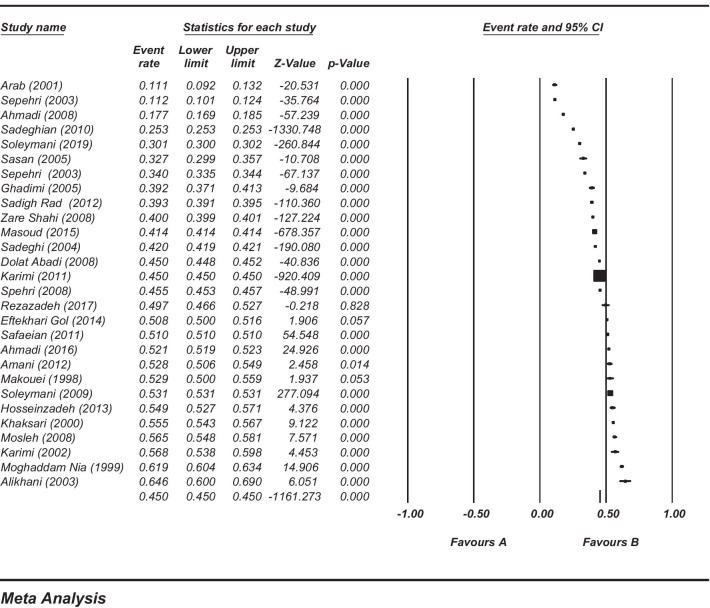
Percentage of antibiotic prescribing in the outpatient settings in Iran

Among the reviewed studies, 32 (59%) ones were conducted in outpatient settings during 1995–2019. In these studies, the unit of analysis was either prescriptions or patients, and the minimum and maximum sample sizes were 441 and 200,000,000, respectively. Most of the studies (75%) did not report the most commonly prescribed antibiotic classes. Penicillin, cephalosporin, macrolides, as well as aminoglycosides were the most commonly prescribed antibiotic classes. Amoxicillin, penicillin, co-amoxiclav, and cefixime were the most frequently prescribed antibiotics (Table 4). Figure 2 shows the meta-analysis results and the percentages of prescribed antibiotics extracted from 27 studies conducted in outpatient settings. The total mean of antibiotic-containing prescriptions was 45% and their median in outpatient settings was 45.25%.

**Table 4:** General characteristics of the studies conducted in outpatient healthcare settings

References	Region	Sample size/unit of analysis	Percentage of antibiotics prescribed	The most commonly prescribed antibiotic classes (from most to least)	The most commonly prescribed antibiotics (from most to least)
Khaksari et al. (1995 and 2000) [63]	Rafsanjan	6895 prescriptions	55.5	NM	NM
Mousavi et al. [64]	Iran (all provinces)	NM (prescription data from all around the country)	45	NM	NM
Makouei et al. [65]	Urmia	1090 prescriptions	53	Penicillin, Sulfonamides, Cephalosporin, Aminoglycosides, Macrolides	Penicillin, Amoxicillin, Ampicillin, Co-amoxiclav, Cloxacillin
Dinarvand et al. [66]	Tehran	NM (prescriptions in 55 pharmacy)	43	NM	NM
Soleymani et al.(1998–2009) [67]	Iran (all provinces)	200 million prescriptions	53.1	NM	NM
Moghaddam Nia et al. [68]	Babol	4000 prescriptions	61.9	NM	NM
Arab et al. [69]	Khuzestan	986 prescriptions	17.1	NM	NM
Cheragh Ali et al. [29]	Iran (100 primary care settings from 5 provinces of Tehran, Fars, Khorasan, Khuzestan, Kermanshah)	NM (prescriptions in 100 primary care settings)	58	NM	Amoxicillin, Procaine Penicillin
Karimi et al. [70]	Savojbolagh	1068 prescriptions	56.8	NM	Penicillin, Azithromycin, Penicillin 6.3.3, Cefixime, Co-amoxiclav
Sepehri et al. [71]	Kerman	45,384 prescriptions	33.95	Penicillin, Cephalosporin, Aminoglycosides	NM
Alikhani et al. [72]	Yasuj	441 prescriptions	64.6	Penicillin, Macrolides, Cephalosporin, Nalidixic-acid, Sulfonamides,	Erythromycin, Amoxicillin, Co-amoxiclav, Penicillin, Cefixime,
Sepehri et al. [73]	Bam	3000 prescriptions	11.2	Penicillin, Cephalosporin, Macrolides, Tetracycline, Aminoglycosides	Amoxicillin, Penicillin G, Cephalexin, Penicillin 6.3.3, Co-trimoxazole,
Bastani et al. (2003–2013) [74]	Iran (all provinces)	59 million prescriptions in each year	53.33	NM	NM
Sadeghi et al. [75]	Khorasan Jonubi	1,423,642 prescriptions	42	NM	Amoxicillin, Cefixime
Ghadimi et al. [76]	East Azarbayjan	2041 prescriptions	39.2	NM	NM
Sasan et al. [77]	Mashhad	1000 infants and children	32.7	NM	Penicillin 6.3.3, Co-trimoxazole, Ampicillin, Gentamicin, Amoxicillin
Masoud et al. (2005–2015) [78]	Kerman	15,784,313 prescriptions	41.42	NM	NM
Dolat Abadi et al. [79]	Sabzevar	167,305 prescriptions	45	NM	Amoxicillin
Ahmadi et al. [80]	Ahvaz	9524 prescriptions	17.7	NM	NM
Sepehri et al. [81]	Bam	297,104 prescriptions	45.5	Penicillin, Cephalosporin	Ciprofloxacin, Metronidazole, Amoxicillin
Zare Shahi et al. [82]	Kerman	410,218 prescriptions	40	NM	NM
Mosleh et al. [83]	Tehran	3420 prescriptions	56.49	NM	NM
Sadeghian et al. [84]	Isfahan	7,999,530 prescriptions	25.31	Penicillin, Cephalosporin, Macrolides, Quinolones, Sulfonamides	NM
Safaeian et al. [85]	Isfahan	7,439,709 prescriptions	51	Penicillin, Cephalosporin, Macrolides, Quinolones, Sulfonamides,	Cefixime, Amoxicillin, Co-amoxiclav, Penicillin 6.3.3, Azithromycin,
Karimi et al. [28]	Iran (all provinces)	85 million prescriptions	45	NM	Amoxicillin
Sadigh Rad et al. [86]	Urmia	269,660 prescriptions	39.29	NM	NM
Amani [87]	Ardabil	2000 prescriptions	52.8	NM	Penicillin 6.3.3, Ceftriaxone, Penicillin 800,000
Hossein Zadeh et al. [88]	Ardabil	2000 prescriptions	54.9	Penicillin, Cephalosporin, Macrolides	Cefixime, Azithromycin, Co-amoxiclav, Penicillin 6.3.3, Amoxicillin,
Eftekhari Gol et al. [89]	Khorasan Razavi	14,189 prescriptions	50.8	Nm	NM
Ahmadi et al. [90]	Kermanshah	352,399 prescriptions	52.1	NM	NM
Rezazadeh et al. [91]	Tehran	1035 prescriptions	49.71	NM	NM
Soleymani et al. [92]	Tehran	455,549 prescriptions	30.1	NM	NM
		Median: 14,189	Median: 45.25	Most prescribed: Penicillin, Cephalosporin, Macrolides, and Aminoglycosides	Most prescribed: Amoxicillin, Penicillin, Co-amoxiclav, and Cefixime
		IQR1: 2000	IQR1: 39.2		
		IQR3: 939,595	IQR3: 53.33

NM: Not Mentioned.

## Discussion

This study aimed at providing an overview of the antibiotic prescribing pattern in Iran, as an example of developing countries. The results of this study showed that the rate of antibiotic prescribing in inpatient and outpatient settings in Iran was 68.2% for patients and 45.25% for prescriptions, respectively. Cephalosporins and carbapenems were the most commonly prescribed antibiotic classes in inpatient settings, while in outpatient settings, they were penicillins, cephalosporins, and macrolides.

Due to overprescribing of antibiotics in Iran, there are few studies using interventions to improve the pattern of antibiotic prescribing. Although there are different potential interventions pertaining to rational antibiotic prescribing, almost all the studies conducted in Iran have used educational interventions for physicians. Many of these interventions had no statistically significant effect on improving antibiotic prescribing pattern. It seems, nowadays, these traditional interventions have little effectiveness rather than electronic interventions. It is predicted that IT-based interventions with the provision of some capabilities such as regularly and automated registration of medications, performance feedback, and a reminder to physicians, and easy access to information at the point of care can help to more rational prescribing medications. Some of the studies [93–96] have shown that IT-based interventions (such as clinical decision support systems (CDSSs), electronic health record (EHR), electronic prescribing, and electronic based feedback on physician’s performance) could improve antibiotic prescribing pattern. Studying IT-based interventions and their effects merits further research.

We found that more than two-thirds of patients received antibiotics in the inpatient settings in Iran (median = 68.2%). The results of a global study showed that antibiotic consumption increased by 65% in 76 countries from 2000 to 2015 (from 21.1 to 34.8 billion DDDs) [97]. The experts from the center for disease control and prevention found that the total rate of antibiotic use in the United States hospitals did not change from 2006–2012, and that more than half of the patients received at least one antibiotic during their hospital stay [98]. The rate of antibiotic prescribing in Iran (68.2%) is similar to that reported in an original study in Nigeria (69.7%) and surpassed the WHO recommended range of 20–26.8% and in some developed countries such as Italy. Moreover, the rate of antibiotic prescribing in Iran is less than that in some developing countries such as Turkey, India, China, Tunisia, and Greece [97, 99]. Thus, since the antibiotic prescribing rate is high in the inpatient settings in Iran, applying interventions to improve that is necessary.

The results of this study showed that nearly half of the outpatients received antibiotics in Iran (median = 45.25% and meta-analysis = 45%). The results of a study by Yin et al. [18] showed that antibiotic prescribing in outpatient centers in China was 50.3%, and almost more than half of the outpatient visits in China resulted in prescribing antibiotics. In addition, Li et al. [26] reported the rate of antibiotic prescribing at URTI outpatient centers in China as 83.7%. The antibiotic prescribing rate is high in the United States as well, and almost 269 million antibiotic prescriptions were dispensed from outpatient pharmacies in 2015. Moreover, 5 out of 6 Americans received an antibiotic prescription in that year [75]. Another study in the United States showed that the mean of antibiotic prescribing per 1,000 patients was 826 cases in 2013 and 2015 in outpatients [100]. The overall rate of antibiotic prescribing in Iranian outpatient settings was higher than in many other undeveloped countries such as Maldives (24%), Bangladesh (31%), DPR Korea (35%), Cameroon (36.71%), Bhutan (41%), and East Timor (43%), but similar to Nepal (44%) and Indonesia (45%), and less than in Myanmar (47%), Sri Lanka (56%), India (62%), and Jordan (78%) in Africa, the Middle East, and East Asia [15, 16, 101]. Since the antibiotic prescribing rate is high in outpatients in Iran, interventions to reduce antibiotic prescribing rate is necessary. While educational interventions had no significant effect on reducing antibiotic prescribing rate in this country, the use of new interventional methods is suggested.

The most common antibiotic classes prescribed in inpatient settings were cephalosporins, penicillins, and carbapenems, while in outpatient settings, they were penicillins, cephalosporins, and macrolides. Furthermore, the most frequently prescribed antibiotics were ceftriaxone and cefazolin in inpatient settings and amoxicillin, penicillin, co-amoxiclav, and cefixime in outpatient settings. Antibiotics classes such as penicillins, cephalosporins, quinolones, and macrolides were the most common antibiotic classes consumed in 76 countries during 2000–2015 [97]. Although consumption of broad-spectrum penicillins, carbapenems, and polymyxins has increased in high, middle, and low-income countries, there are some differences in the consumption of antibiotic classes. For example, cephalosporins consumption has increased in low and middle income countries, while it has declined in high income countries [97]. Also, in outpatient settings in the USA, the most commonly prescribed antibiotics in 2018 were azithromycin, amoxicillin, ciprofloxacin, and cephalexin [100]. Some of the most prescribed antibiotics in this study such as amoxicillin and cefazoline were placed in the access group and cefexime, ceftriaxone and vancomycin were placed in the watch group based on Access, Watch, Reserve (AWaRe) classification of antibiotics by WHO [102]. Despite the high use of some antibiotic classes such as carbapenems, quinolones, and cephalosporins, particularly the third generation of broad-spectrum antibiotics, they should be used with caution. These antibiotic classes have a high potential to cause antimicrobial resistance or side effects; however, their consumption has increased rapidly in low and middle income countries, while it has decreased in high income countries. [97, 103]. Thus, based on the obtained results, it seems necessary to change the antibiotic consumption patterns in Iran.

### Implications

Our results may help increase the awareness and knowledge about the antimicrobials use and identifying areas of overuse in this country. Moreover, Iranian health policymakers could develop a national plan to improve the clinical application of antibiotics and consider use of recommended IT-based interventions.

### Strength and limitations

This study is the first to describe the prevalence of antibiotic prescriptions in Iran. It also provides an overview of interventions taken to improve antibiotic prescriptions in this country. However, the Persian search engine is limited but we conducted several search strategies such as searching Google Scholar, hand-searching, and searching reference lists of the included studies. We did exclude different kind of studies: studies describing antibiotic prescribing in surgery, dentistry, and burn patients (because of the necessity of antibiotics); studies that reported antibiotic prescription without the frequencies that we sought (because they reported DDD, DID, DBD scales); and studies that assessed only special classes of antibiotics such as vancomycin, imipenem, aminoglycosides, meropenem, ciprofloxacin (because they do not provide a comprehensive picture of antibiotic prescribing).

## Conclusion

This study showed that antibiotic prescribing rate in both inpatient and outpatient settings in Iran surpasses the WHO recommendations and exceeds that in many other countries. Moreover, this study revealed that traditional educational interventions showed no significant effect on reducing antibiotic prescribing rate. In order to decrease antibiotic prescribing by physicians, IT-based interventions such as electronic feedback on physicians’ performance, electronic prescribing, and clinical decision support systems may hold promise.

## Supplementary Information

**Additional file 1: Attachment 1.** Percentage of antibiotic prescribing in all wards of hospitals in Iran. **Attachment 2.** Percentage of antibiotic prescribing in pediatrics wards of hospitals in Iran. **Attachment 3.** Percentage of antibiotic prescribing in ICU wards of hospitals in Iran.

## Data Availability

The data generated and analyzed during this study are available from the corresponding author on reasonable request.
