# Supplementary material for: Antibiotic prescribing in inpatient and outpatient settings in Iran: a systematic review and meta-analysis study
Source: Antimicrob Resist Infect Control. 2021 Jan 14;10:15. doi: 10.1186/s13756-021-00887-x (PMC7809737; doi:10.1186/s13756-021-00887-x)
Supplement: Supplementary file 1 — Additional file 1: Attachment 1. Percentage of antibiotic prescribing in all wards of hospitals in Iran. Attachment 2. Percentage of antibiotic prescribing in pediatrics wards of hospitals in Iran. Attachment 3. Percentage of antibiotic prescribing in ICU wards of hospitals in Iran. [file 13756_2021_887_MOESM1_ESM.docx]

**Attachments**

**Attachment 1. Percentage of antibiotic prescribing in all wards of hospitals in Iran**

**Attachment 2. Percentage of antibiotic prescribing in pediatrics wards of hospitals in Iran**

**Attachment 3. Percentage of antibiotic prescribing in ICU wards of hospitals in Iran**
